# Autologous Transplantation of Adult Mice Spermatogonial Stem Cells into Gamma Irradiated Testes

**Published:** 2012-08-31

**Authors:** Morteza Koruji, Mansoureh Movahedin, Seyed Javad Mowla, Hamid Gourabi, Shahram Pour-Beiranvand, Ali Jabbari Arfaee

**Affiliations:** 1. Cellular and Molecular Research Center, Tehran University of Medical Science, Tehran, Iran; 2. Department of Anatomical Sciences, School of Medical Sciences, Tehran University of Medical Sciences, Tehran, Iran; 3. Department of Anatomical Sciences, School of Medical Sciences, Tarbiat Modares University, Tehran, Iran; 4. Department of Genetics, School of Basic Sciences, Tarbiat Modares University, Tehran, Iran; 5. Department of Genetics, Reproductive Biomedicine Research Center, Royan Institute for Reproductive Biomedicine, ACECR, Tehran, Iran; 6. Department of Radiation Oncology, Shohada-E-Tajrish Hospital, Tehran, Iran

**Keywords:** Spermatogenesis, Stem Spermatogonia, Gamma-Irradiated, Auto Transplantation

## Abstract

**Objective:**

We evaluated structural and functional changes of fresh and frozen-thawed adult mouse spermatogonial stem cells following auto-transplantation into gamma-irradiated testes.

**Materials and Methods:**

In this experimental research, the right testes from adult mice (n=25) were collected, then Sertoli and spermatogonial cells were isolated using two-step enzymatic digestion, lectin immobilization and differential plating. Three weeks after cultivation, the Bromodeoxyuridine (BrdU)-labeled spermatogonial cells were transplanted, via rete testis, into the other testis of the same mouse, which had been irradiated with 14Gy. The mice were transplanted with: fresh cells (control 1), fresh cells co-cultured with Sertoli cells (control 2), the frozen-thawed cells (experimental 1) and frozen-thawed cells co-cultured with Sertoli cells (experimental 2). The morphological changes between different transplanted testes groups were compared in 8 weeks after transplantation. The statistical significance between mean values was determined by Kruskal Wallis and one-way analysis of variance in efficiency of transplantation.

**Results:**

The statistical analysis revealed significant increases in the mean percentage of testis weight and normal seminiferous tubules following spermatogonial stem cells transplantation in the recipient'fs testes. The normal seminiferous tubules percentage in the co-culture system with fresh cells and frozen-thawed groups were more than those in non-transplanted and fresh cell transplanted groups (p≤0.001).

**Conclusion:**

Our results demonstrated that spermatogonial stem cells in the colonies could result sperm production in the recipient’s testes after autologous transplantation.

## Introduction

Spermatogenesis is a highly organized and complex process, characterized by self-renewal of undifferentiated spermatogonial stem cells (SSCs) and production of differentiated daughter cells to provide a continual supply of spermatozoa (1). The sub-population of testicular stem cells is believed to be very small (comprising 1 in 3333 cells of adult mouse testis) (2). However, an *in vitro* system that supports SSCs survival and proliferation is useful for enhancement of stem cell number and efficient transplantation (3). Several culture systems have already been developed for *in vitro* maintenance and propagation of spermatogonial cells of various species (4-10). It has previously been reported that somatic cells can support the proliferation of isolated adult (11) or prepubertal mouse(12, 13), porcine (5), human (14, 15) and bovine (8, 16) SSCs, for short or long term period. Based on several other reports, SSCs can be cryopreserved for prolonged periods of time (1, 17, 18) and maintain their capability of establishing spermatogenesis after transplantation in the recipient’s testis (7, 19, 20).

Since 1994, when the first germ cell transplantation technique has been developed (21, 22), several research groups employed the technique for various applications (23). One of the uses of SSCs transplantation is to identify the functional SSCs in a germ cell suspension and to compare SSCs numbers after various treatments or culture periods (24, 25).

Although homologous and heterologous transplantation have been reported in mice and several other species (26-31), autologous transplantation has just been investigated in monkey (32), pig (33) and bovine (31). A complete restoration of spermatogenesis has not been observed yet in monkey and pig; whereas, a limited restoration of spermatogenesis has been reported in bovine after autologous transplantation. These studies clearly have showed that the efficiency of transplantation is highly dependent on the phylogenic distance between the donor and the recipient species. Yet, an autologous transplantation of adult mouse SSCs has not been reported in mice.

For most children diagnosed with cancer, cure is a likely outcome (34). During the recent years, treatment successes in young boys with cancer have led to substantial increase in survival rate (35) . Cytotoxic drug therapy and radiotherapy for eradicate cancer cells can damage spermatogenesis and lead to temporary or permanent infertility in young prepubertal patients. Theoretically isolation , propagation and cryopreservation of SSCs from the patients undergoing chemotherapy or radiotherapy could provide a source of endogenous SSCs for possible auto-transplantation in the future. Base on the theory, the main aim of the current study was to evaluate structural and functional changes of fresh and frozen-thawed adult mouse SSCs following autologous transplantation within gamma-irradiated testes.

## Materials and Methods

### Animals

Male adult National Medical Research Institute (NMRI) mice (aged=6-8 weeks old; n=25), derived from the original stocks obtained from Razi Laboratory (Tehran, Iran), were maintained under the standard conditions with free access to food and water at the Animal Facility of Tarbiat Modares University. The experimental study was conducted in accordance with the guidelines of the National Research Council (affiliated to the Tarbiat Modares University).

### Germ cell collection, cryopreservation and confirmation

Adult NMRI mice (6-8 weeks old, n=5 per each group) were hemi-castrated to allow autologous transplantation. Accordingly, the right testis from each mouse was collected for preparation of the cell suspension following enzymatic digestions and purification steps. After overnight differential plating, the floating cells were collected and either cultured or cryopreserved as described elsewhere (11, 18). Briefly, after decapsulation, the right testis was minced separately and suspended in Dulbecco's Modified Eagle Medium (DMEM; Gibco, Paisley, UK) which contained 0.5 mg/ml collagenase/dispase, 0.5 mg/ml Trypsin, and 0.05 mg/ml DNase, for 30 minutes (with shaking and a little pipetting) at 37℃. All enzymes were purchased from Sigma Company (Sigma, St. Louis, MO, USA). Then, the most of interstitial cells, spermatozoa and some spermatids cells were removed by washing in DMEM medium. Second digestion step was performed in DMEM media by adding fresh enzymes solution into the seminiferous cord fragments. After cell separation and filtration through 70-µm nylon filter, the collected cells were used for the removal of spermatocyte cells. The spermatocyte cells were removed using a procedure described by van Pelt et al. (36). The Sertoli cells were also isolated using a procedure described by Scarpino et al. (37). Briefly, Petri dishes with a diameter of 60 mm or flasks were coated with a solution of 5 µg/ml of datura stramonium agglutinin (DSA; Sigma, USA) in phosphate-buffered saline (PBS) at 37℃ for 1 hr. Then, the coated plastic dishes were washed three times with DMEM containing 0.5% Bovine Serum Albumin (BSA; Sigma, USA) The mixed population of the cells obtained by enzymatic digestion was placed on lectin-coated dishes and incubated for 1 hour at 32℃ in a humidified atmosphere of 5% CO_2_. After incubation, the non-adherent cells were collected by washing with handling medium twice. Immediately after cell isolation, cell viability was assessed.

The isolated cells were cryopreserved using a procedure described by Izadyar et al. with some modification (17). Cell suspensions in 0.5ml aliquots (6×10^6^ cells per mL) were prepared. Then, an equal volume of 2×concentrated freezing medium was added drop-wise to the Eppendorf vial containing the cell suspension during a period of 10-15 minutes and gently mixed by inverting the vial; afterward, a sample was taken for viability assessment. The freezing media were based on DMEM supplemented with 10% (v/v) FCS, 1.4M DMSO and 0.07 M sucrose. For freezing, 1.8-ml cryovials vials (Nunc, Denmark) containing 1ml of cell suspension in freezing medium were placed in an insulated (polystyrene) container at-70℃ for at least 1 day, then plunged into liquid nitrogen. The cells were thawed by swirling in 38℃ water bath for 2 minutes. The contents of the vial was transferred to a tube and diluted slowly by adding two volumes of DMEM supplemented with 10% fetal calf serum (FCS). Then, the cells were pooled and centrifuged at 2000×g for 5 minutes, the supernatant was removed, and the pellet was resuspended in DMEM/FCS. A sample was taken for viability assessment, and the remainders of the cells were used for culture experiments.

The frozen-thawed spermatogonial cells were cultured in two groups: simple culture (experimental 1) and co-culture with Sertoli cells (experimental 2). In addition, fresh spermatogonial cells were cultured and processed along-side frozen-thawed cells, as two control groups: simple culture (control 1) and co-culture with Sertoli cells (control 2). In co-culture groups, a monolayer of Sertoli cells was previously prepared, then spermatogonial cells were co-cultured on top of them (11, 37). The cells were incubated at 32 ℃ in a humidified atmosphere of 5% CO_2_ and the presence of 10% FCS. The cells were grown for 3 weeks, then the identity of the cultured cells was confirmed by detection of vimentin in the Sertoli cells and OCT-4. C-KIT immunocytochemistry as well as alkaline phosphatase activity of the obtained colonies were also performed as described elsewhere (11).

### Recipient testis preparation, BrdU incorporation, and transplantation procedure

The left testes were irradiated with 14Gy of ^60^Co γ-ray from cobalt therapy machine (Shohada-E-Tajrish Hospital) at 10-12 weeks of age as previously described (38). Absence of endogenous spermatogenesis were tested and confirmed within irradiated recipient’s testes, at the time of transplantation (4 weeks after radiation treatment). The culture of experimental mouse spermatogonial cell colonies from right testis were treated with BrdU [Bromodeoxyuridine (5-bromo-2'-deoxyuridine)] (Sigma, USA) followed by isolating the Sertoli cells by DSA lectin immobilization. Then the remaining labeled-cells were transplanted into the seminiferous tubules of the other testis of the same mouse via rete testis. Briefly, the animals were anesthetized using ketamine 10% and xylazine 2% (Alfasan, Woerden, Netherlands), then 105 donor spermatogonial cells in 10µl DMEM and 2µl trypan blue (to assess the efficiency of cell transplantation) were injected into the seminiferous tubules of the left testis. Presence of transplanted cells was assayed by immunohistochemical detection of BrdU-incorporated cells according to the manufacturer’s instructions (Sigma, USA) (11). The left testis which was irradiated without transplantation in other hemicastrated mice was considered as the non-transplanted group.

### Efficiency of transplantation

Two parameters used to determine the efficiency of transplantation are as follows: Testicular mass weight and morphological changes in recipient’s testes.

The transplanted mouse testes were examined 8 weeks after transplantation. The testes were weighted, fixed in 4% paraformaldehyde, dehydrated, and embedded in paraffin. The 5µm-thick sections were then immunostained with the primary antibody (1:100) against BrdU (Sigma, USA).

For assessment of morphological changes in each recipient’s testis, almost 300 randomly selected tubular profiles (round or nearly round) were determined and classified based on the percentage observed cases into 3 types: normal seminiferous tubule with sperm, normal seminiferous tubule without sperm, and depleted seminiferous tubule.

### Statistical analysis

Results are expressed as mean±SD. The statistical significance between mean values was determined by Kruskalwallis and one-way analysis of variance in efficiency of transplantation. A value of p.0.05 was considered as significant.

## Results

### Effect of cryopreservation on viability rate after thawing

Viability rate of cells after isolation process and mixing by freezing media were 92.8 ± 6.03% and 85.5 ± 5.7%, respectively. These findings demonstrate that freezing media does not have a significant effect on viability rate. Only 68.4 ± 10.2% of the frozen cells survived after cryopreservation. The viability rate was significantly (p≤0.001) influenced by the freezing and the thawing procedure.

### Colonization assay of transplanted cells

**Fig 1:**
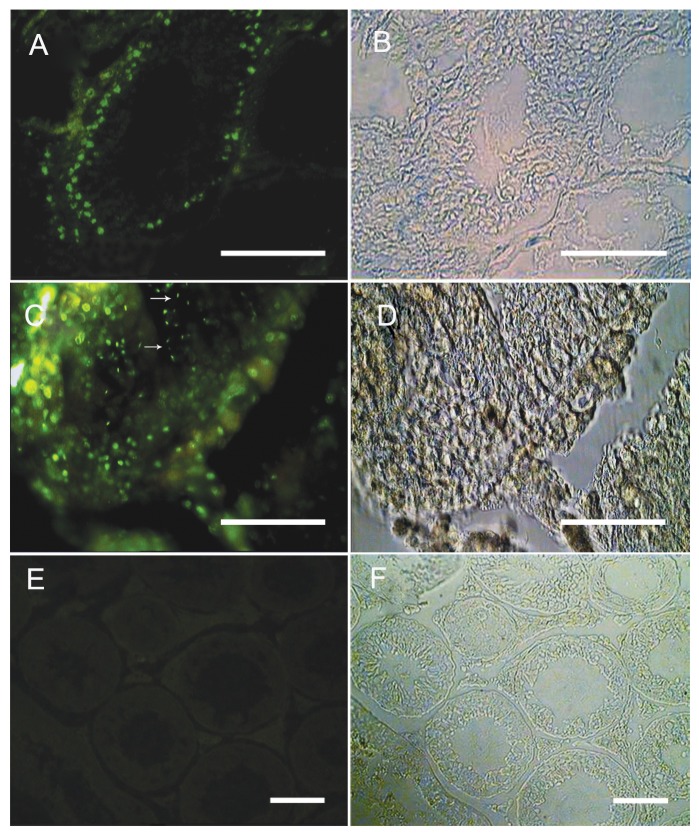
Auto-transplantation of adult mouse spermatogonia resulted in spermatogenesis in recipient testis, The spermatogonial cells were labeled with BrdU *in vitro* and transplanted into the recipient testes via rete testis. Donor-derived spermatogonial cells were traced in the recipient testes (A) two weeks. (C) 8 weeks after transplantation elongated spermatids were detected within the testis (Arrows). (E) Control group without adding primary antibody. The cells showing nuclear BrdU staining were considered as transplanted cells. (B, D, F) Phase contrast photographs. (Bar=50µm).

To trace the fate of spermatogonial cells after transplantation, BrdU was added to the cell culture 24 hours before transplantation. To confirm the identity of the propagated SSC cells and their colonization status within the testis, 105 BrdU-labeled cells of different culture groups were grafted into the seminiferous tubules of the recipient’s left testis. Two weeks after transplantation, the labeled cells were traced in the base of seminiferous tubules of recipient’s testes. Eight weeks after transplantation, elongated spermatids showed BrdU-labeled nuclei were detected within the testis (Fig 1-F).

### Efficiency of transplantation

#### Testicular mass weight

During the 4^th^ weeks after irradiation, about 82.6 ± 1.9 of tubules were depleted of spermatogenesis and only a small percentage of the tubules contained spermatocytes in the irradiated testes. By this time, the mean of irradiated testis mass of mice, 10-12 weeks old, was about 0.025 ± 0.005 g/testis in comparison to those of non-irradiated mice which was 0.130 ± 0.010 g/testis (38). A significant increase (3X) in the testis mass was observed after autologous transplantation of germ cells co-cultured with the Sertoli cells group in comparison to non-transplanted group. The mean of testis mass after autologous transplantation increased up to 0.06 ± 0.008 g/testis at the time of examination (18-20 weeks old animals); while, the mean of irradiated testis mass at non-transplanted group (12 weeks post-irradiation) was about 0.02 ± 0.003 g/testis. Overall, the testis mass after autologous transplantation in all groups was increased significantly in comparison to the non-transplanted group (p≤0.05) (Table 1).

### Morphological changes in the recipient testes

**Fig 2:**
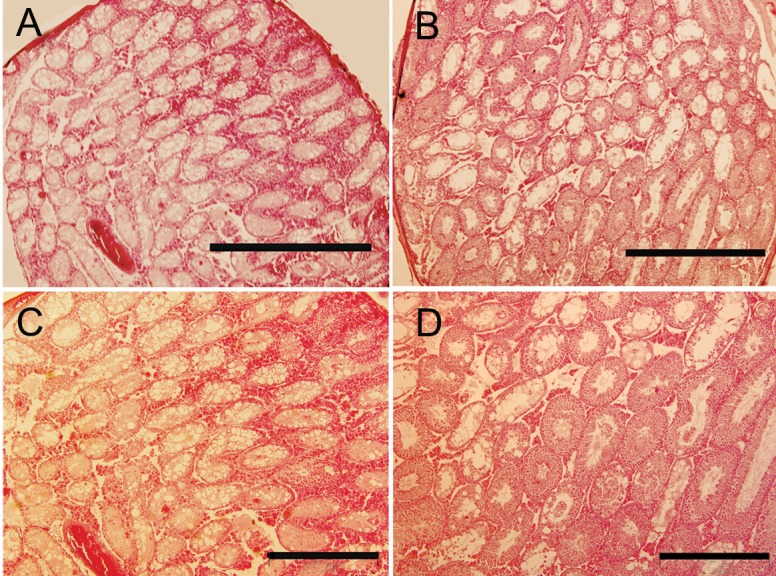
(A, C) A non-transplanted testis in 12 weeks after irradiation, the majority of the seminiferous tubules still contained only Sertoli cells. (B, D) Eight weeks after the auto-transplantation, more differentiated germ cell types are seen in seminiferous tubules. (Bar=1000 µm in A, B; Bar=500 µm in C, D).

Examination under a light microscopy revealed spermatogenesis in some tubules in the non-transplanted mice (Fig 2A-D). A significant difference was observed in testis histology between the non-transplanted and all autologous transplanted groups, especially the one co-cultured with Sertoli cells transplanted groups (Table 1). Percentage of the normal and depleted tubules in the fresh cells and frozen-thawed co-cultured with Sertoli cells transplanted group (control 2 and experimental 2) were 80.68 ± 6.20, 10.95 ± 5.85 (control 2); and 64.16 ± 9.33, 19.94 ± 6.02 (experimental 2), respectively. In contrast, only 10.9 ± 4.2% of the tubules were normal and 77.7 ± 9.8% of the tubules were depleted in the non-transplanted group. The depleted tubules in the transplanted groups were altered commensurate with normal tubules (Table 1). There was no significant difference in the percentages of normal tubules without sperm among all groups (Table 1).

**Table 1:** 


Efficiency parameters	Non-transplanted	Control 1	Control 2	Experimental 1	Experimental 2

Testicular mass (g)	0.024 ± 0.003^*^	0.046 ± 0.005	0.066 ± 0.008^*^	0.043 ± 0.010	0.048 ± 0.007
Depleted tubules (%)	77.6 ± 9.8^*^	48.6 ± 7.9^*^	10.95 ± 5.85	25.35 ± 3.91^**^	19.94 ± 6.02
Normal tubules with sperm (%)	10.9 ± 4.2^*^	33.0 ± 2.9^*^	80.68 ± 6.20^*^	65.78 ± 3.24	64.16 ± 9.33
Normal tubules without sperm (%)	11.4 ± 6.1	18.4 ± 10.1	8.37 ± 2.54	8.87 ± 1.13	15.90 ± 10.73


Results are expressed as mean±SD. Results from five separate experiments were used for all groups. * Significant difference vs. all groups in the same row (p≤0.001). ** Significant difference vs. control 2 in the same row (p≤0.001).

## Discussion

In this study, we demonstrated that autologous transplantation of adult fresh and frozen-thawed spermatogonial cells after cultivation can resume spermatogenesis in a recipient’s testis.

 There are several critical factors to achieve a successful transplantation of SSCs, including: a. the number of SSCs in donor cell suspension for autologous transplantation, b. suitable recipient’s testes, c. an efficient transplantation procedure (8, 16) and d. proximity in phylogenesis.

To increase the number of SSCs in donor cell suspension, we co-cultured adult germ cells with Sertoli cells and obtained colony formation. Our previous results showed that the number of the colonies and their diameters in the fresh and frozen-thawed germ cells co-cultured with Sertoli cells was increased significantly in comparison with the control group during 2 weeks cultivation. After that, the number of colonies in frozen groups declined significantly (18) and it seemed that our culture system couldn’t support the frozen-thawed SSCs. On the other hand, we had to transplant SSCs after 3 weeks culture. As we know, transplantation results are dependent on cultural findings.

Previous studies have shown that co-cultures of mouse and bovine spermatogonia with Sertoli cells (8, 11, 16, 18, 39) or using residual testicular somatic in mouse (13, 40) and human (14) culture improved SSCs proliferation. Further works demonstrated when donor testis cells were transplanted without enrichment for SSCs, only 10% of tubules were colonized with donor cells (22). In our study, increase in the number of tubules containing donor-derived spermatogenic cells confirmed the enriched number of fresh and frozen-thawed SSCs by co-culturing with Sertoli cells *in vitro* and the improvement of colony formation before transplantation.

Various reports have shown that the species, dosage and regimen, the age of the subject, and the kind of radiation might have significant effects on the long-term outcome of the testes transplantation model (17, 31, 41-43). Based on our previous studies, local single γ -irradiation (14 Gy) leads to the depletion of endogenous spermatogenesis in the seminiferous tubules of the recipient’s testis (38), suggesting its suitability as a model for transplantation (11).

Direct injection of donor cells into rodent seminiferous tubules is possible via the efferent ducts, which is feasible with mouth pipette and a stereomicroscope. We transplanted SSCs into the testis of recipient using these simple tools. The BrdU-labeled injected cells within recipient’s testes resumed spermatogenesis which was evident by nuclear BrdU staining. In this study, both freshly and cryopreserved SSCs were functional and produced more advanced germ cells in the recipient’s testes. It was probably due to the phylogenetic proximity between the donor and the recipient species. Our results were consistent with previous studies. Honaramooz et al. demonstrated that after homologous transplantation in goats, the recipient goats became sexually mature and produced the transgenic offspring (28). Anjamrooz et al. showed that heterologous transplantation of neonate spermatogonial cells after co-culture with Sertoli cells increased the number of spermatozoa in the epididymal lumen of recipient mice (9). Also, Kanatsu-Shinohara et al. showed that live offspring could result from spermatozoa produced after transplantation of frozen–thawed mouse germ cells (1). Meanwhile, SSCs did not produce more advanced germ cells in xenogeneic spermatogonial transplantation (26, 30, 44).

The efficiency of transplantation co-cultured with Sertoli cell groups (fresh and frozen-thawed cells) and just frozen-thawed cells were higher compared to the simple culture groups. The increased numbers of SSCs in the transplanted cell population can result in better efficiency of transplantation. In our previous studies, we demonstrated that a co-culture system with Sertoli cells could increase *in vitro* colony formation or the number of adult fresh and frozen-thawed spermatogonial cells (11, 18, 39). Transplantation of large number of SSCs enhance their homing into tubules and the level of donor cell colonization (7). We proposed that it also may result to restore the spermatogenesis in the arrested tubules. In previous autologous and homologues transplantation have been observed a significant increase in the testis mass (3X) and in the percentage of tubules containing spermatogenic cells (≤80%) (31). Also, sperms arising from transplanted donor germ cells were capable of fertilization *in vivo* (20) and *in vitro* (21, 28, 45). On the other hand, the infertile recipient animals are likely to become fertile with donor-derived gametes when at least 50% of the tubules contain donor-derived spermatogenic cells (33, 46). In our study, although the testis mass increased and more than %50 of tubules showed donor-derived spermatogenic cells in the fresh and frozen-thawed cells co-culture transplanted groups, still the recipient mice were infertile. The previous studies have showed that the restoration of fertility requires higher SSCs numbers. At least, it takes 3 months for immature and 5 months for adult after transplantation (7, 46, 47).

## Conclusion

Finally, our results might have some clinical implications, mostly in application of auto-transplantation of SSCs for the restoration of spermatogenesis in cancer patients undergoing intense chemo- and radiotherapy. This provides new methodology in handling spermatogenesis, particularly in transplants.
